# The Symptoms and Clinical Diagnosis of Hiatus Hernia

**Published:** 1952-04

**Authors:** Ronald Belsey

**Affiliations:** Consultant Thoracic Surgeon, South-West Region


### THE SYMPTOMS AND CLINICAL DIAGNOSIS OF HIATUS HERNIA

BY

RONALD BELSEY, M.S., F.R.C.S.

*Consultant Thoracic Surgeon, South-West Region*

Herniation of the cardiac end of the stomach through the oesophageal hiatus of the diaphragm, and the resulting disorganization of the normal sphincter mechanism at the cardia, is a common disease that can cause distress at any period of the patient's life from early infancy onwards. Most cases fall into two groups: children under the age of seven, and adults in their fifth to seventh decades. The lowest incidence is amongst adolescents and young adults but patients who first complain to their doctors in the later decades will often volunteer the information that they " have had trouble all their lives, but worse recently ". The sexes are affected about equally up to the age of fifty, when the incidence amongst females rises steeply above that amongst males. Some patients date their symptoms from a pregnancy; others remain free from distress till late in life, a fact difficult to correlate with the growing belief that the majority of these hernias are congenital in origin, and suggesting rather that factors other than the mere presence of part of the stomach in the chest may be responsible for the symptoms, or for the complications usually causing the symptoms.

The reason why the pathological significance of hiatus hernia and the misery it can cause the patient have occasioned so little clinical interest in the past, has been the frequent co-existence of other lesions, abdominal, cardiac, or mediastinal, which have tended to mask the hernia as the culprit. The patient's distress was attributed to the small cluster of gall-stones, the hypotonic stomach, or to some occult cardiac condition vaguely suspected. Even when improvements in radiological technique began to bring these hernias to light, there was still no move to assign to them any responsibility for the patient's symptoms and the traditional text-book myth of their innocence continued to bewitch the clinician. As recently as 1947 Radloff and King in reporting on fifty cases of hiatus hernia stated: " In sixty-eight per cent concomitant disease was found and was often responsible for the symptoms. Only thirty per cent had symptoms that could be attributed to the hernia. Surgery was necessary in only three cases." They concluded: " Diaphragmatic hernia is usually an insignificant finding, and is unusually asymptomatic."

This opinion is at variance with the experience of most clinics in this country, where at least fifty per cent of cases, and in children up to eighty per cent, show oesophageal ulceration, stenosis, or some other accepted indication for surgical treatment. Moreover, in thirty per cent of these cases the gall-bladder, appendix, or other organs considered responsible for the concomitant disease and the patient's symptoms have already been removed. For years cholecystectomy has been the standard treatment in patients suffering from hiatus hernia. Harrington

(1948) reports that in every one of his cases an average of three previous diagnoses had been made, and in ten per cent previous abdominal operations had been undertaken.

It is an accepted fact that this abnormality may be compatible with perfect health and complete freedom from symptoms but the considered opinion of numerous authors suggests that only twenty per cent are asymptomatic; this figure may require considerable modification when a statistically significant number of proven cases have been followed up throughout their normal span.

Hiatus hernia first came under suspicion as a prolific cause of subjective distress when the pioneer work of Allison (1948) implicated this condition in the aetiology of such obvious disabilities as gross fibrous stenosis of the oesophagus, repeated gastro-intestinal haemorrhage, anaemia, and duodenal obstruction, complications which can hardly be assigned to gall-stones, chronic appendicitis and diverticulosis of the duodenum or colon.

One of the most difficult problems for the clinician confronted by a patient with a radiologically demonstrated hiatus hernia is the assignment of the patient's various symptoms to it or to other underlying lesions which may be present in the heart or gall-bladder for example, and only consideration of the differential diagnoses and a complete and thorough investigation to exclude these alternative and possibly co-existing lesions can save the clinician from an embarrassing error, and the patient from an unnecessary operation. The problem may have been simplified by the previous removal of most of the other organs usually incriminated but this particular form of " diagnosis by exclusion " is not to the patient's advantage. Brick (1949) found associated lesions capable of causing symptoms in one in every four patients in his series of 308 cases. Clerf, Shallow, Putney and Fry (1950) list the errors in diagnosis that had previously been made in 35 of their 110 cases. They then state that an unspecified number of their cases, not included amongst the 35, had multiple lesions—" cholecystitis with or without gall-stones, coronary artery disease, duodenal ulcer or traction diverticulum ". Gilbert, Dey and Rall (1946), in search of associated lesions in support of the theory that a vagal reflex and oesophageal spasm are the cause of the hernia, found 27 upper abdominal and thoracic lesions amongst their 48 cases.

In theory at any rate, and in practice whenever the supply of X-ray film renders it possible, every patient with a proven, uncomplicated, hiatus hernia should have a cholecystogram, a complete barium investigation of the stomach and duodenum, and a cardiological check-up. When the hernia is already complicated by oesophagitis, chronic ulceration, or stenosis, one is on safer ground in assuming its responsibility for at least the major portion of the patient's distress. By the same token, the patient with symptoms of so-called " gall-bladder dyspepsia " should have a barium swallow in the prone head-low position as well as a cholecystogram.

When the presence of multpile lesions has been revealed, common sense and clinical acumen, whatever these may be, must dictate which, if any, require treatment, and the order of precedence. The abdominal surgeon will probably remove the gall-bladder first; the thoracic surgeon will be more interested in the hiatus hernia, but it is probable that each will be wrong with equal frequency. Only from the follow-up clinic will " the light " eventually emerge, and the time and labour spent in objective post-operative surveys, preferably conducted by a third disinterested party, will shorten the period of medieval obscurity that surrounds this and so many other problems in surgery.

The co-existence of gastric ulceration and hiatus hernia presents a further problem in that acute high lesser curve ulceration appears to be common in the patient with an hiatus hernia, and the intermittent haemorrhage that occurs may come from this source rather than from the oesophageal ulceration. In short, there may be a close aetiological relationship between the two conditions and on occasion simple repair of an hiatus hernia has led to the prompt healing of a chronic lesser curve ulcer refractory to all forms of medical treatment. There appears to be a closer relationship between these two conditions than can be explained by mere coincidence.

The hazards of diagnosis are well illustrated by the following case. A man of fifty-nine years, suffering from the characteristic symptoms of hiatus hernia, but rather more dyspnoea than usual for a man of his age, was referred to a thoracic unit for oesophagoscopy and further investigation. He had previously been seen by a cardiologist on account of his dyspnoea and the heart pronounced as normal. On the day before admission he dropped dead in a cinema. Autopsy revealed an hiatus hernia and gross aortic valvular stenosis.

The clinical picture presented by a case of hiatus hernia is fairly typical, and the following description by Allison (1951) cannot be bettered:

A woman of fifty-nine years of age complains that for six years she has suffered from intense burning pain behind the lower part of the sternum, which rises up towards, or even into, the neck. It may spread into the jaw, the ear or the hard palate, or radiate through to the back between the shoulder blades, or down the arm. It comes on especially when she exerts herself stooping forward, as in washing the floor, bending over the wash-tub, poking the fire or fastening her shoes. It wakes her in the middle of the night, especially if she is sleeping on her back or her right side, and she seeks relief from what she describes as an agonizing pain by sitting upright and taking a few sips of water, milk or alkaline mixture. She says that her throat usually feels dry and burning. When she swallows she may be conscious of the passage of food down the gullet, it may cause a feeling of soreness and may sometimes lodge towards the lower end of the sternum, causing pain which is immediately relieved as the bolus passes into the stomach. If she bends forward after a meal, food or sour fluid rises into her throat and has to be swallowed again. Her husband says that for belching she takes the first prize. Four years ago she was thought to have cholecystitis, but removal of either a normal or abnormal gall-bladder did not cure her. Radiography of her stomach and duodenum shows no evidence of ulcer. She has tried all the advertised stomach medicines with only temporary relief, and has finally been told that " the nerves of her stomach have been upset by the change of life ". This story, with minor variations, occurs often enough in the hospital out-patient department to deserve more notice and better treatment.

Pain, heartburn, dysphagia, and postural regurgitation are the commonest symptoms of this condition.

Pain, epigastric and substernal, is the symptom from which the patient most frequently seeks relief. The pain radiates upwards and may extend down the arms; it is nearly always aggravated by stooping forward or by lying flat at night and most patients discover for themselves the relief to be obtained by sleeping propped up in bed on several pillows at night. When oesophageal ulceration is present the pain may also extend backwards between the shoulder blades and tends to be more severe, but even in the absence of oesophagitis pain is often the presenting symptom. Fortunately pain is the symptom most frequently and completely relieved by surgical reduction and repair of the hernia.

Chronic heartburn and acid regurgitation are common, and result from the incompetence of the normal sphincter mechanism at the cardia which follows its divorce from the right crus of the diaphragm. The occurrence of chronic heartburn is virtually pathonomonic of an hiatus hernia and is seen in few other conditions. It does not necessarily indicate the existence of oesophagitis and is just as common in the uncomplicated hernia.

Regurgitation is usually promoted by changes in posture, such as stooping or lying down, but may occur without any warning nausea quite suddenly at the start of, or during, a meal. The patient may have to rush from the table to vomit, but can then return and finish the meal without further difficulty. No regurgitation may occur for weeks or months, then suddenly it returns. The very uncertainty of this occurrence and the embarrassment involved prompts the sensitive patient to shun all social engagements. Some say they are afraid to eat, but the appetite remains good and weight loss is uncommon.

Dysphagia occurs in two forms. When oesophagitis is present there may be pain on swallowing, especially of hot fluids, and also the sensation of a hold up in the lower substernal region. The patient may localize this sensation in the region of the cervical oesophagus, and a diagnosis of globus hystericus inevitably follows. When secondary fibrous stenosis of the lower third complicates chronic reflux oesophagitis the symptoms become those of organic oesophageal obstruction—greater distress and prompt regurgitation following attempts to swallow, the regurgitation of frothy mucous between meals and first thing in the morning, and rapid wasting of the patient. The stenosis may develop at any time between infancy and old age. An infant of four months has recently been admitted to Frenchay Hospital with almost complete obstruction of the lower oesophagus, necessitating an emergency gastrostomy. In older patients the obstruction may be of short duration and progressive, prompting a clinical diagnosis of carcinoma. Why severe ulceration and stenosis should develop quite suddenly in a patient of sixty or seventy, or even eighty, remains one of the mysteries of the natural history of this disease. It is probable that those cases said in the past to have been cured of oesophageal carcinoma by a gastrostomy and a course of X-ray therapy were in fact examples of these inflammatory strictures; a sufficient number have now been excised and examined histologically to exclude the possibility of atypical scirrhus malignant disease, unproven by biopsy, as a cause of the stenosis.

Haemorrhage may also occur in two forms. In the presence of oesophagitis the vomit may be streaked with blood. Frank haematemesis, sometimes massive, also occurs but probably from a different cause; the acute ulceration of the lesser curve of the stomach already mentioned is the more likely source. Secondary anaemia is frequently seen but not constantly in association with oesophagitis or gastric ulceration, as one would expect, suggesting that some other factor is involved. In children on the other hand, the relationship between oesophagitis and anaemia is much more constant.

Various atypical symptom complexes may be seen. There is one type of pain associated with a hernia, and undoubtedly caused by it, which may resemble the pain of angina. The patient complains of acute praecordial pain or distress, coming on in spasms, and radiating up to the left or both sides of the neck, and down the left arm; but the pain usually follows a meal and is not related to exercise. Nuzum (1947) reported an incidence of twenty-five patients with hiatus hernia in 100 private patients diagnosed as having angina pectoris; Gilbert (1948) found an incidence of eighteen, or 17 per cent, with angina-like pain in 107 patients with hiatus hernia.

Two patients in whom the presenting symptom was acute pain behind the right ear due to a vagal reflex with pain reference to the distribution of its auricular branch, have been investigated at Frenchay. The co-existence of other more characteristic symptoms—heartburn and postural regurgitation—led to the diagnosis.

Another interesting group of patients present with pulmonary symptoms—chronic productive cough and dyspnoea due to pulmonary fibrosis and bronchiectasis, the result of nocturnal regurgitation, and recurring bouts of acute aspiration pneumonitis. A doctor recently sought relief by surgery because of the frequency with which he was awakened at night choking in his own vomit.

Other cases are first seen as a result of an acute catastrophe. Two patients in each of whom there was a history of the typical symptoms, but too mild to take them to their doctors, were admitted to hospital moribund and died shortly afterwards, one with an acute ulcer at the cardia which had perforated into the hernial sac, the other with gangrene of the intra-thoracic portion of the stomach due to torsion.

In the great majority of cases the history and symptoms present a clinical picture which can now be regarded as characteristic of hiatus hernia and which enables a provisional diagnosis to be made with fair accuracy. Any patient complaining of chronic heartburn, intermittent regurgitation, dysphagia, and substernal or epigastric pain aggravated by stooping or lying flat should be subjected to the routine investigations necessary to confirm the diagnosis: a barium swallow examination in the prone head-low position, and oesophagoscopy.

In considering the differential diagnosis, the possible coincidence of two or more common conditions must be envisaged. The conditions most likely to cause confusion are cholecystitis, peptic ulceration of the stomach and duodenum, and other causes of dysphagia, such as carcinoma of the oesophagus or stomach. Fortunately there are available methods of special investigation which render the differentiation of these conditions a relatively simple process. No longer should it ever be necessary to resort to such thoroughly unsatisfactory diagnoses as "chronic appendicular dyspepsia, cyclical vomiting, gastrostaxis, abdominal migraine, or the functional abdomen".

No mention has been made of the clinical picture presented by the child suffering from this condition; he complains of nothing but is ill, and often desperately so, with vomiting, anaemia, wasting, and acute starvation due to oesophageal obstruction. He may run a persistent pyrexia, due to the inflammation of the oesophagus or the chronic aspiration pneumonitis. In neither the child nor the adult are there any characteristic physical signs that can be elicited on clinical examination; it is their very absence that sometimes leads to the diagnosis.

In conclusion, mention should be made of four simple tests that may assist in the clinical diagnosis of hiatus hernia prior to resort to special methods of investigation:

(i) The effect of posture and the relief obtained by the patient when encouraged to sleep propped up;

(ii) The failure of alkalis to relieve appreciably the pain and regurgitation, due to the mechanical factors involved;

(iii) The absence of epigastric tenderness in the presence of epigastric pain;

(iv) A history of previous abdominal operations and their failure to relieve the patients' symptoms.

### REFERENCES

Allison, P. R., (1948). *Thorax* **3**, 20.
Allison, P. R. (1951). *Surg. Gynec. Obstet.*, **92**, 419.
Brick, I. B. (1949). *Mississipi Valley M. J.*, **71**, 2.
Clerf, L. H., Shallow, T. A., Putney, F. J., and Fry, K. E., (1950). *J. Amer. Med. Assoc.*, **143**, 169.
Gilbert, N. C., Dey, F. L., and Rall, J. E. (1946). J. *Amer. Med. Assoc.*, **132**, 132.
Gilbert, N. C. (1948). *M. Clin. N. Amer.*, **32**, 213.
Harrington, S. W. (1948). *Surg. Gynec. Obstet.*, **86**, 735.
Nuzum, F. R. (1947). *Amer. Heart J.*, **33**, 724.
Radloff, F. F., and King, R. L., (1947). *Gastroenterology*, **9**, 249.

